# Sharply Contrasting Chemotypes Coincide with Aggression and Divergence in Cryptic African Carpenter Ant Populations

**DOI:** 10.1007/s10886-026-01732-4

**Published:** 2026-06-22

**Authors:** Marius Pohl, Tobias Laukamp, Marius Caspers, Selina Paczkowski, Jan Buellesbach

**Affiliations:** 1 Institute for Evolution and Biodiversity, University of Münster, Hüfferstraße 1, Münster, 48149 Germany; 2 Evolutionary Chemical & Sensory Ecology, Life Science Systems, Technical University of Munich, Hans Carl-von-Carlowitz-Platz 2, Freising, D-85354 Germany

**Keywords:** Cuticular hydrocarbons, Chemical taxonomy, aggression behavior, Genetic barcoding, Hymenoptera, Formicidae, *Camponotus*

## Abstract

Cryptic differentiation poses a significant challenge to taxonomy, particularly in morphologically conserved lineages whose divergence is not immediately apparent. In eusocial insects like ants, where species boundaries are often maintained through chemical rather than morphological differentiation, integrative methodologies are essential for resolving taxonomic complexity. Here, we present compelling evidence for the recent divergence of African carpenter ants initially identified as *Camponotus maculatus* into two distinct sub-populations. This divergence is supported through an integrative approach combining chemical, behavioral, and genetic analyses. Most strikingly, we identify two sharply contrasting chemotypes characterized by categorically different cuticular hydrocarbon (CHC) profiles that unambiguously separate the investigated ant populations. Concordantly, behavioral assays reveal that worker ants consistently exhibit aggression toward individuals with opposing chemotypes, while displaying affiliative behaviors toward shared chemotypes irrespective of colony affiliation. Genetic barcoding further corroborates these findings, indicated by phylogenetic clusters largely corresponding with the two chemotypes. Our results highlight the primary role of chemical differentiation within morphologically indistinguishable populations to resolve cryptic sub-population structures. These findings also provide valuable insights into potential early-stage chemical differentiation mechanisms in eusocial insect populations.

**Supplementary Information:**

The online version contains supplementary material available at https://doi.org/10.1007/s10886-026-01732-4.

## Introduction

Since the time of Aristotle’s pioneering animal classification efforts around 350 BCE, taxonomy has relied heavily on visible morphological traits to characterize distinct taxa (Aristotle and Schneider 1878, Gill 1911; Groves and Harding 2003). However, external morphology alone may not always provide sufficient resolution to capture the full extent of biodiversity, particularly in lineages where morphological convergence, stasis, or plasticity obscure true evolutionary relationships (Padial et al. 2010; Steenwyk and King 2024). With the advent of molecular systematics and integrative taxonomic methods, it has become increasingly clear that cryptic differentiation, i.e., genetically distinct sub-populations, lineages or species, is far more common than previously recognized (Struck et al. 2018; Hending 2025). Cryptic taxa have now been documented across most lineages on the tree of life, challenging classical taxonomic frameworks based on morphology alone and prompting a re-evaluation of the criteria by which taxonomic boundaries are defined (Jörger and Schrödl 2013; Li and Wiens 2023). With approximately one million described extant species, insects comprise around half of all currently identified animal taxa (Li and Wiens 2023; Bánki et al. 2024). However, including cryptic species, recent estimates on the true biodiversity of insects exceed 20 million species in total (Li and Wiens 2023). Given the alarming and persistent decline in insect biodiversity, despite their tremendous importance in almost all ecosystems on Earth (Verma et al. 2023), the lack of recognition of cryptic insect taxa poses a particular challenge (Scudder 2009; Vodă et al. 2015; Raven and Wagner 2021). Accurate taxonomic identification is crucial for most conservation initiatives, integrated protection programs, and ecological studies (Hull et al. 1977; Balakrishnan 2005; Li and Wiens 2023).

This is particularly evident in ants (Hymenoptera: Formicidae), where taxonomic groups with subtle morphological differences and extensive polymorphisms are frequently poorly resolved when relying solely on morphology-based taxonomy (Lucas et al. 2002; Heinze et al. 2005; Schlick-Steiner et al. 2006). With their complex social systems and extensive reliance on chemical communication, ant lineages frequently exhibit evolutionary divergence that may not be directly and visibly reflected in external morphology (Emery and Tsutsui 2013; Hartke et al. 2019; Sprenger et al. 2021). An example of the complexity in ant taxonomy and divergence can be found within the genus *Lasius*, where initially only 16 distinct Palearctic species have been described based on morphology (Wilson 1955). However, following taxonomic concepts first proposed by Bickford et al. (2007), integrating genetic divergence with classic morphological characteristics, a total of 42 species were identified, with 29% classified as cryptic species (Seifert 2009). Similar investigations have been carried out in ant genera such as *Formica*, *Myrmica*, *Nylanderia*, *Temnothorax* and *Pachycondyla*, e.g., the discovery of three distinct *Pachycondyla* species formerly described as a single species (*P. villosa*) (Seifert 1993, 2003; Lucas et al. 2002). The efficiency of morphological taxonomy depends on the availability of fast and reliable discrimination techniques based on distinctive phenotypic characteristics, which may be limited in cryptic ant taxa (Seifert 1995, 2008; Moder et al. 2007). In such cases, the integration of morphology with complementary approaches can enhance the resolution of potentially cryptic ant lineages and contribute to a more realistic estimate of their diversity (Jörger and Schrödl 2013; Emerson 2025).

Chemical taxonomy has emerged as a promising complementary tool in taxonomic studies that proves to be particularly efficient in organisms heavily relying on different modalities of chemical signaling (Bagnères and Wicker-Thomas 2010; Van der Meer 2019). Eusocial insects like ants exhibit multiple chemical communication systems governing their behavior and social organization, allowing them to coordinate activities, identify conspecifics and colony members, and respond to threats (Hölldobler 1995; Greenfield 2002; Jackson and Ratnieks 2006). The most prevalent and well-studied components of ant chemical communication are cuticular hydrocarbons (CHCs), long-chained lipids comprising complex profiles that constitute a major part of the waxy outer layer of an insect’s epicuticle (Blomquist and Bagnères 2010; Kather and Martin 2015; Sprenger and Menzel 2020). They have been shown to convey a wide variety of differential chemical information, including reproductive status, social hierarchy and nestmate as well as species affiliation (Sharma et al. 2015; Leonhardt et al. 2016; Smith and Liebig 2017). The latter two are of particular importance, as CHCs constitute key mediators of intra- and interspecific ant aggression, allowing for the distinction of either nestmates from non-nestmates or con- from heterospecific individuals (Ozaki et al. 2005; Lalzar et al. 2010; Kidokoro-Kobayashi et al. 2012). Increased levels of aggression are usually directed towards individuals whose CHC profiles have been assessed as either non-nestmate or heterospecific, revealing a perceptive capability to detect even minute chemical differences (Errard et al. 2006; Guerrieri et al. 2009; Watanabe et al. 2023). Concordantly, CHC profiles have also frequently been used as chemotaxonomic tools that can complement morphology-based identifications, particularly in taxa where species boundaries are difficult to distinguish, and therefore hold considerable potential to disentangling cryptic species complexes (Bagnères and Wicker-Thomas 2010; Kather and Martin 2015).

**Fig. 1:**
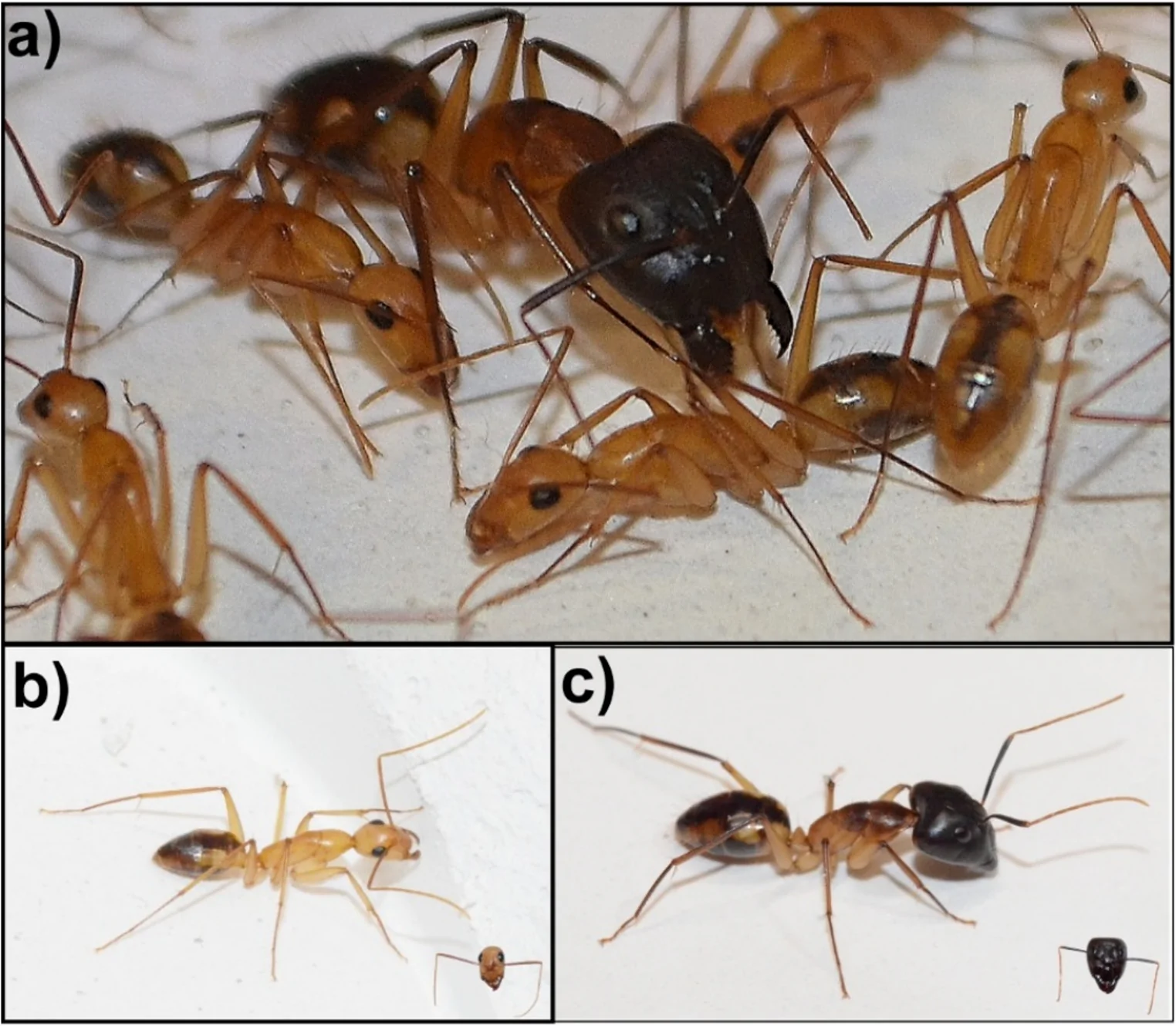
**a** Picture of a major and several minor workers in our experimental colonies. Note the distinct size difference. **b** Single image of an isolated minor worker, with its head profile displayed on the bottom right corner. **c** Single image of an isolated major worker, with its head profile displayed on the bottom right corner. All images were captured with a Nikon D5100 digital camera equipped with an AF-S Nikkor 18–55 mm 1:3.5-5.6G objective (Nikon Precision Europe GmbH, Langen, Germany)

The carpenter ant genus *Camponotus* Mayr, 1861 constitutes one of the most diverse and species-rich ant genera, including an estimated 1000 species with additional 500 subspecies belonging to 45 subgenera (Bolton 2021). This extensive species richness, coupled with high levels of distinctive intraspecific polymorphisms and geographic variation, renders disentangling this genus one of the most complex and challenging tasks in animal taxonomy (Bolton 2003; Brady 2003; Seifert 2019a). *C. maculatus* Fabricius, 1782, a particularly widely distributed *Camponotus* species ranging from Africa across the Middle East to Asia (McArthur and Leys 2006; Clouse et al. 2015), is characterized by a high degree of color variation and morphological polymorphisms (Donisthorpe 1915; Baroni Urbani 1972). *C. maculatus* colonies also exhibit a distinct worker dimorphism, differentiating its worker caste into major and minor workers, primarily distinguished by body size and task allocation (Fig. 1). Minor workers with body sizes ranging from 6 to 10 mm primarily maintain the nest, perform brood care and forage, while the larger major workers (13–16 mm) predominantly defend the nest, essentially acting as a soldier caste (McArthur and Leys 2006; Ferguson et al. 2023).

In this study, we focus on *Camponotus* colonies collected within the same habitat at the Ivory Coast in West-Africa that were preliminarily identified as *C. maculatus* based on their common morphology. We are combining 

i. chemical divergence based on colony CHC profiles, 

ii. behavioral aggression assays of the workers castes within and between colonies, and 

iii. mitochondrial genetic barcoding. Our integrative methodologies reveal the presence of two sharply distinct CHC chemotypes separating the sympatric ant populations and predicting unambiguous behavioral aggression between but not within them, independent of colony affiliation. Mitochondrial barcoding further supports these uncovered chemotypes, indicating incipient divergence between them. By integrating these chemical, behavioral and molecular approaches, we were able to differentiate the investigated carpenter ant populations and refine our understanding of intricate cryptic diversity within this ant lineage. In addition, the differentiation in CHC profiles, combined with the apparent capability of the workers to distinguish between them, highlights the value of chemical taxonomy as a complementary tool in systematics and population divergence studies.

## Materials and Methods

### Collection and Maintenance of Study Organisms

Multiple mated queens of *Camponotus maculatus* were collected between April and June in the years 2018 and 2019 from the Comoé National Park at the Ivory Coast in Western Africa. Queens were sampled during their nuptial flight period using a blacklight at the same location close to the Comoé Research Station (8° 46′ 11″ N, 3° 47′ 21″ W). Afterwards, the queens were transferred to climate-controlled cabinets (model AR-95 L/3, CLF PlantClimatics GmbH, Wertingen, Germany), where they were subsequently housed in plastic boxes (20 cm x 20 cm x 9 cm, Dino AG, Berlin, Germany), containing a plastered floor, and fed twice a week with honey water and cockroach legs (*Blaptica dubia*). Box lids had four ventilation holes (Ø = 4 cm) and were covered with metal mesh, and a petri dish covered with red plastic foil within the boxes simulated a nesting area. Founding colonies were maintained at a day / night cycle of 30 °C / 25 °C for 12 h each at 50% relative humidity for at least a year to let them grow to a sufficient colony size (> 2000 workers) prior to carrying out the described experiments (see below). All required collection and export permits were provided by the Directeur Général of the Office Ivoirien des Parcs et Réserves (OIPR), Côte d’Ivoire (N°018/MINEDD/OIPR/DZ). Workers from representative colonies were visually inspected and used for a qualitative morphological assessment under 10–80 x magnification using a Leica M205 C microscope (Leica Camera AG, Wetzlar, Germany). Morphological characters taken into consideration were head shape; eye size and position; clypeus shape; scape length in relation to head length; petiole shape as well as abundance of standing pilosity on head, mesosoma and gaster. This initial assessment was intended to identify apparent, conspicuous morphological differences. No quantitative morphometric analyses were conducted due to the notorious difficulty in reliably assessing divergence in this ant taxa based solely on morphology(Baroni-Urbani 1972; Bolton 2003, 2021).

### Chemical Analyses

Major (*n* = 34) and minor workers (*n* = 34) from different colonies were freeze-killed for at least one hour at -25 °C. Each ant was then individually placed in a 2 ml crimp vial (Agilent, Santa Clara, California, USA) and extracted in 250 µl of MS-pure hexane (UniSolv, Darmstadt, Germany) for 10 min on an orbital shaker (IKA KS 130 Basic, Staufen, Germany). Extracts were then transferred with a disposable Pasteur glass pipette (Brand GmbH + Co, Wertheim, Germany) to a 250 µl conical insert (Agilent, Santa Clara, California, U.S.A.) and evaporated under a constant flow of CO₂. The dried extract was then resuspended with 10 µl MS-pure hexane containing 7.5 ng/µl of n-dodecane (EMD Millipore Corporation, Burlington, Massachusetts, USA) as an internal standard. Five µl of the reconstituted extract were injected into a gas chromatograph (GC: 7890B) simultaneously coupled to a flame ionization detector (FID: G3440B) and a tandem mass spectrometer (MS/MS: 7010B, all provided by Agilent Technologies, Waldbronn, Germany) with a PAL autosampler system operating in electron impact ionization mode with 70 eV. The split/splitless injector was operating at 300 °C in pulsed splitless mode at 20 psi until 0.75 min with the purge flow set to split vent at 50 ml/min at 0.9 min. The GC system was equipped with a fused silica column (DB-5MS ultra inert; 30 m x 250 μm x 0.25 μm; Agilent J&W GC columns, Santa Clara, CA, USA), and helium was used as carrier gas at a constant flow rate of 1.8 ml/min. The FID operated at a temperature of 300 °C and utilized nitrogen as the make-up gas with a flow rate of 20 ml/min, and hydrogen as the fuel gas with a flow rate of 30 ml/min. The column was split at an auxiliary electronic pressure control (Aux EPC) module, with one portion leading to the FID detector through a deactivated fused silica column piece (0.9 m x 250 μm) at a flow rate of 0.8 mL/min, and the other portion leading to the mass spectrometer through another deactivated fused silica column piece (1.33 m x 250 μm) at a flow rate of 1.33 ml/min. The temperature program for the column started at 60 °C and was held for 2 min, before heating up to 200 °C with a rate of 60 °C per minute, followed by a subsequent increase of 4 °C per minute until reaching the final temperature of 325 °C, which was held for 5 min. CHC peak detection, integration, quantification, and identification were performed using the Quantitative Analysis MassHunter Workstation Software (Version B.09.00 / Build 9.0.647.0, Agilent Technologies, Santa Clara, California, USA). CHCs were identified based on their retention indices, diagnostic ions, and mass spectra derived from the total ion count (TIC) chromatograms, whereas their quantification was achieved by simultaneously obtained flame ionization detector (FID) chromatograms, which is the optimal method for hydrocarbon quantification (Agilent Technologies, Waldbronn, Germany, pers. comm.). To determine the absolute quantities of CHCs (in ng), each compound was calibrated using three replicates of a dilution series based on the closest eluting *n*-alkane from a C21-40 standard series (Merck, KGaA, Darmstadt, Germany) with concentrations of 0.5, 1, 2, 5, 10, 20, and 40 ng/µl, respectively.

### Aggression Assays

To investigate behavioral differentiation between the two chemically distinct *Camponotus* chemotypes, we conducted a series of standardized 1-on-1 aggression assays under controlled conditions. The primary objective was to assess whether workers from different chemotypes exhibit increased inter-colonial aggression compared to interactions between colonies of the same chemotype. Therefore, we included both major and minor workers from four distinct colonies (two per chemotype) in all possible inter- and intra-colonial combinations. For each unique combination, ten replicates were performed, resulting in a total of 200 individual aggression assays. Ants were randomly selected from their respective colonies and caste groups to avoid sampling bias. Each individual was marked on the dorsal mesosoma with a small dot of colored Edding paint (Edding Vertrieb GmbH, Wunstorf, Germany) to allow identification during interactions without interfering with their behavior. Following marking, ants were placed individually into clean petri dishes (100 × 20 mm; Diagonal GmbH & Co. KG, Münster, Germany) and allowed to acclimate for 10 min. This period minimized stress and ensured that subsequent behaviors were not influenced by immediate handling or novelty of the arena. Aggression assays were subsequently conducted by gently introducing both ants into a disposable petri dish (100 × 20 mm; Diagonal GmbH & Co. KG, Münster, Germany) under standardized conditions identical to those used for colony maintenance (see above). All aggression assays were recorded for 3 min with a digital camera (Canon EOS 70D, Tokyo, Japan) and evaluated blindly to both colony identity and chemotype by randomizing and reassigning ant marking colours between trials. Behavioral interactions were scored using a modified scale adapted from Krapf et al. (2019). The scale includes eight discrete levels ranging from − 4 (strong affiliative behaviors, such as reciprocal allogrooming) to + 4 (intense aggression, including prolonged biting and gaster flexing; see Table 1 for full scoring scheme).

**Table 1:** Aggression assay scoring scoring table for behavioural assays conducted between major and minor workers from representative colonies for the identified chemotypes

score	behavioural indicator	description of observed behaviours
4	fighting	prolonged aggression, locking mandibles onto a body part, direct spraying of formic acid
3	pseudo-bites	Indication of biting or spraying with formic acid, usually accompanied by gaster curling
2	threatening	mandible flaring (opening of mandibles), trembling
1	avoidance	direct avoidance of contact with the other ant
0	neutral	no interactions
-1	acceptance	close proximity (≤ 1 mm), direct contact
-2	antennation	antennal contact between ants (< 2 s)
-3	allogrooming	reciprocal cleaning
-4	trophallaxis	Exchange of food or fluids through mouth-to-mouth or anus-to-mouth feeding

### Mitochondrial DNA Barcoding

DNA was extracted from the head and thorax of individual *C. maculatus* minor ants using a modified Chelex extraction protocol (Walsh et al. 1991). Each sample was placed in a sterile tube with two metal beads, 100 µl of 1 × TE solution (10 mM Tris-HCL containing 1 mM EDTA * Na_2_, pH 8.0) and 2 µl of Proteinase K (10 mg/ml). The solution was ground in a Mixer Mill (Retsch GmbH, Haan, Germany) for 40 s at 28 Hz. Then, 100 µl of Chelex solution (Bio-Rad Laboratories GmbH, Neuried, Germany) was added and incubated at 57 °C for 1 h, with 60-second agitation every 3 min at 800 rpm on an Eppendorf Thermomixer. Samples were centrifuged at 14,000 rpm for 10 min, and 100 µl of the supernatant was transferred to a sterile 1.5 ml Eppendorf tube and stored at -20 °C.

Polymerase chain reaction (PCR) was performed using 1 µl DNA solution, 2 µl of 5 × reaction buffer, 1 µl of 25 mM MgCl_2_, 0.6 µl of dNTPs (1.25 mM), 0.2 µl of each primer (20 mM), 0.06 µl of GoTaq DNA polymerase (Promega GmbH, Walldorf, Germany), and 4.94 µl distilled water. PCR conditions included initial denaturation (3 min at 94 °C), followed by 38 cycles amplification (94 °C for 60 s, 45 °C for 60 s, 72 °C for 90 s) and a final elongation step (5 min at 72 °C). Reactions were performed on an Eppendorf Thermal Cycler (Eppendorf SE, Hamburg, Germany). Universal primers were used to amplify the mitochondrial cytochrome oxidase subunit I gene (COI, Folmer 1994). PCR products were purified with Exonuclease I and Shrimp Alkaline Phosphatase (ExoSAP-IT™, Thermo-Fisher Scientific, Dreieich, Germany). Five µl PCR product was mixed with 0.5 µl Exonuclease I and 0.25 µl Shrimp Alkaline Phosphatase, incubated at 37 °C for 15 min, 85 °C for 15 min, and cooled to 4 °C.

For Sanger sequencing, 5 µl purified DNA was mixed with 5 µl (5 mM) COI universal primer. The sequencing was performed by Eurofins Genomics Germany GmbH, Ebersberg ,Germany, and sequences were quality-checked using BioEdit 7.2.5 (Hall et al., 2011). Raw COI sequences were edited manually, and primer sequences were removed prior to the alignment. Sequence alignment was performed using the Clustal Omega multiple sequence algorithm (Sievers et al. 2011) with default settings implemented though the EMBL-EBI web server (https://www.ebi.ac.uk/jdispatcher/msa/clustalo). The resulting alignment consisted of approximately 640 base pairs and was subsequently used for phylogenetic reconstruction. Phylogenetic relationships among the tested colonies were tested using the Maximum Likelihood (ML) approach implemented in IQ-TREE v3.1.2 (Minh et al. 2020). The best-fitting nucleotide substitution model was selected using ModelFinder (Kalyaanamoorthy et al. 2017) according to the Bayesian Information Criterion (BIC), resulting in the selection of the TIM2 + F+G4 model (Transition Model 2 with empirical base frequencies and gamma-distributed rate heterogeneity across sites). Branch support was assessed using 10.000 ultrafast bootstrap replicates (UFBoot; Hoang et al. 2017) and 10.000 SH-like approximate likelihood ratio test replicates (Sh-aLRT, Guindon et al. 2010). A sequence of *Camponotus floridanus*, obtained from the NCBI databank (accession number: AY334397.1., https://www.ncbi.nlm.nih.gov/), was used as an outgroup within the same genus to root the phylogeny. The resulting phylogenetic tree was visualized and edited using FigTree v1.5.0.

### Data Visualization and Statistical Analysis

Discriminant analyses (DA) were conducted using the R package “MASS” (Venables and Ripley 2002) to statistically assess chemical divergence based on total CHC profile differences between all investigated ant colonies and castes. Prior to analysis, absolute CHC peak areas (in ng) were standardized by dividing each individual peak area by the total area of the corresponding individual chromatogram, yielding relative abundances. This normalization controlled for body size–related differences in absolute CHC quantities between minor and major workers, as the latter consistently exhibited higher absolute CHC amounts (Fig. S1). To visualize the extent of separation between the investigated groups based on their relative CHC profile differences, the first three discriminant functions from the DA were plotted simultaneously using the R package “scatterplot3d”. For subsequent analyses conducted within each uncovered distinct chemotype, separate DAs were performed, and the first two respective discriminant functions were plotted to reveal caste- and colony-level differentiation within each chemotype.

The statistical significance and strength of group separation in each DA was evaluated using approximated Wilk’s λ statistics. Additionally, for the chemotype-specific DAs, the random forest–based feature selection algorithm “Boruta” (Kursa and Rudnicki 2010) was employed to identify individual CHC compounds most strongly contributing to caste- and colony-level separations.

Differences in absolute CHC amounts, both per compound class and in total, were analyzed separately for minor and major workers using Mann–Whitney U tests, followed by sequential Benjamini–Hochberg corrections for multiple comparisons. Aggression assay data were analyzed by summarizing the tested colonies according to chemotype and then assessing significant differences between the average scale scores of within vs. between chemotype pairings with Benjamini-Hochberg corrected Mann-Whitney U tests as well, separately for major and minor workers. All statistical analysis was performed with R (Version 4.3.1) in R Studio (Version 2023.09.1 + 494) (R Core Team 2024).

## Results

### Chemical Divergence of *C. maculatus* CHC Profiles

**Fig. 2:**
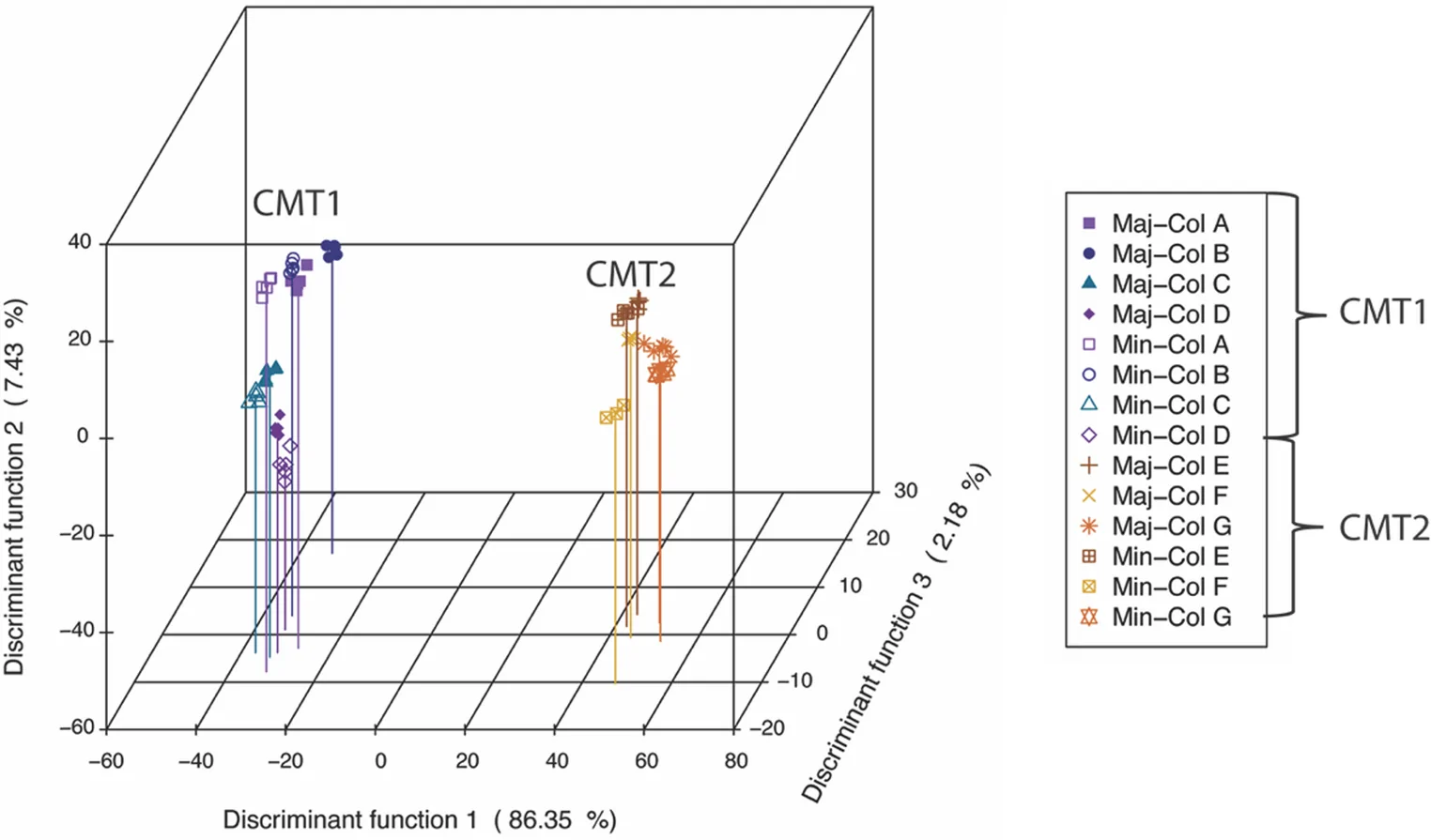
Discriminant analysis (DA) showing the chemical divergence between 68 CHC profiles of major and minor workers from seven investigated colonies with the first three discriminant functions simultaneously plotted in three dimensions. All groups were significantly differentiated from each other (Wilk’s λ < 0.0001, *p* < 0.0001), the variation each function explains is indicated in percentages. Colony affiliations as well as worker castes are indicated by different colors and symbols

Cuticular hydrocarbon (CHC) profiles were analyzed from a total of 68 individual ants, including both major and minor worker castes (*n* = 34 for each), sampled from seven colonies. CHC compounds were quantified and identified using gas chromatography coupled with mass spectrometry (GC-MS), and their relative abundances were subjected to a discriminant analysis (DA) to assess whether chemical signatures reliably differentiate between castes, colonies, and broader chemotypic groupings. The discriminant analysis revealed a highly significant overall separation among groups based on their CHC profiles (approximated Wilks’ λ = 0, χ² = 949, *p* < 0.001). Visualization of the first three discriminant functions in a three-dimensional plot showed a clear and consistent segregation of the colonies into two major clusters (Fig. 2), which we classified as chemotype 1 (CMT1; colonies A–D) and chemotype 2 (CMT2; colonies E–G). The first discriminant function, which accounts for the majority of the observed variation (86.35%), effectively separates the two chemotypes. The second and third discriminant functions explain an additional 7.43% and 2.18% of the total variance, respectively, and appear to mainly distinguish more subtle caste- and colony-specific signatures within the two different chemotypes. A full list of the identified CHC compounds, along with their relative abundances per caste and colony, is provided in Table S1.

### Distinction of *C. maculatus* Chemotypes

**Fig. 3:**
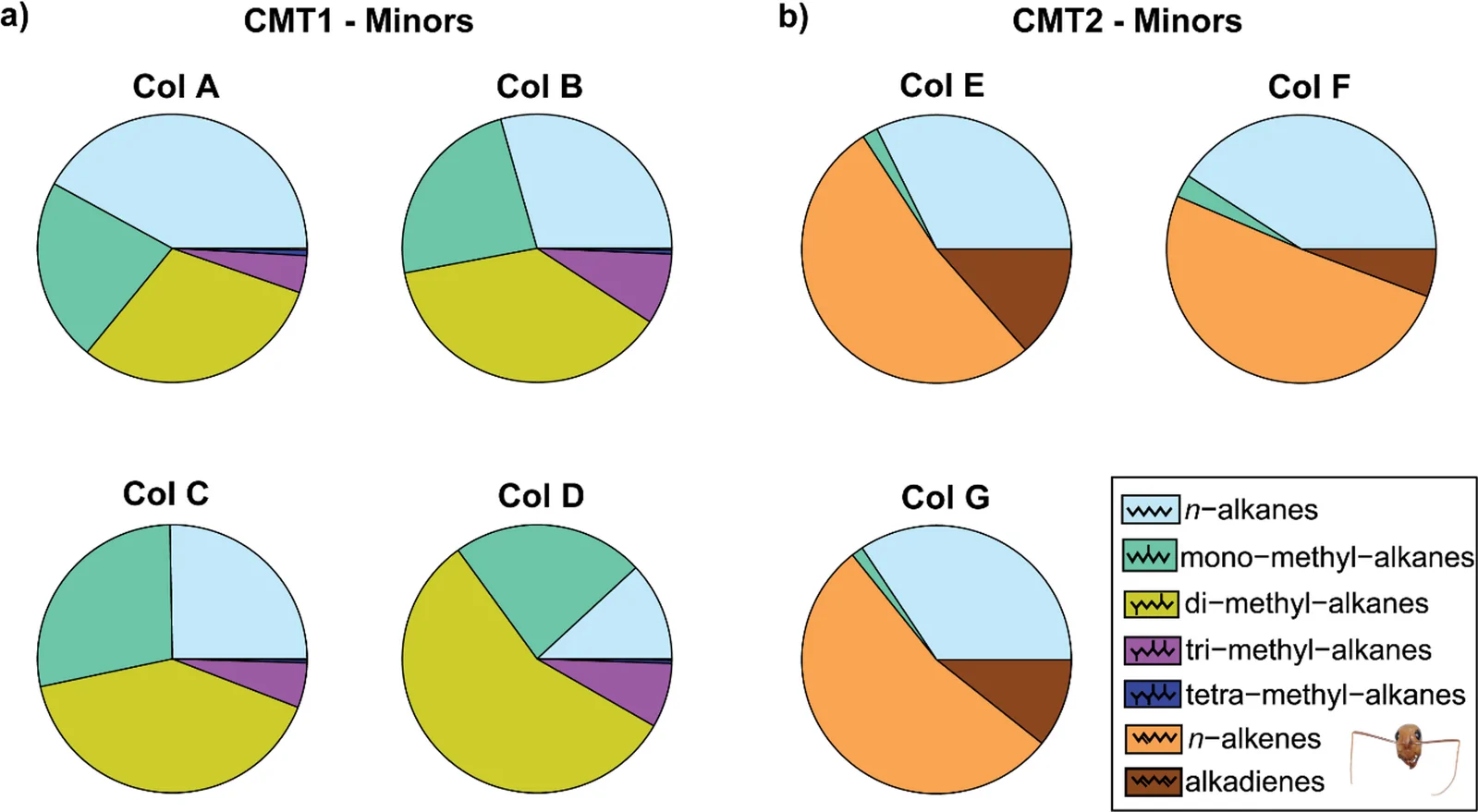
**a** Relative proportions of CHC compound classes detected in minor workers from colonies A-D, subsequently referred to as chemotype (CMT) 1. **b** Relative proportions of CHC compound classes detected in minor workers from colonies E-G attributed to CMT2. Proportions were obtained by dividing individual absolute CHC compound quantities (in ng) by the total area of the corresponding chromatogram and then summarizing each identified compound according to their respective compound class, separately for colony and worker caste (see below), yielding relative abundances. Different colors indicate the distinct CHC compound classes as displayed in the legend

**Fig. 4:**
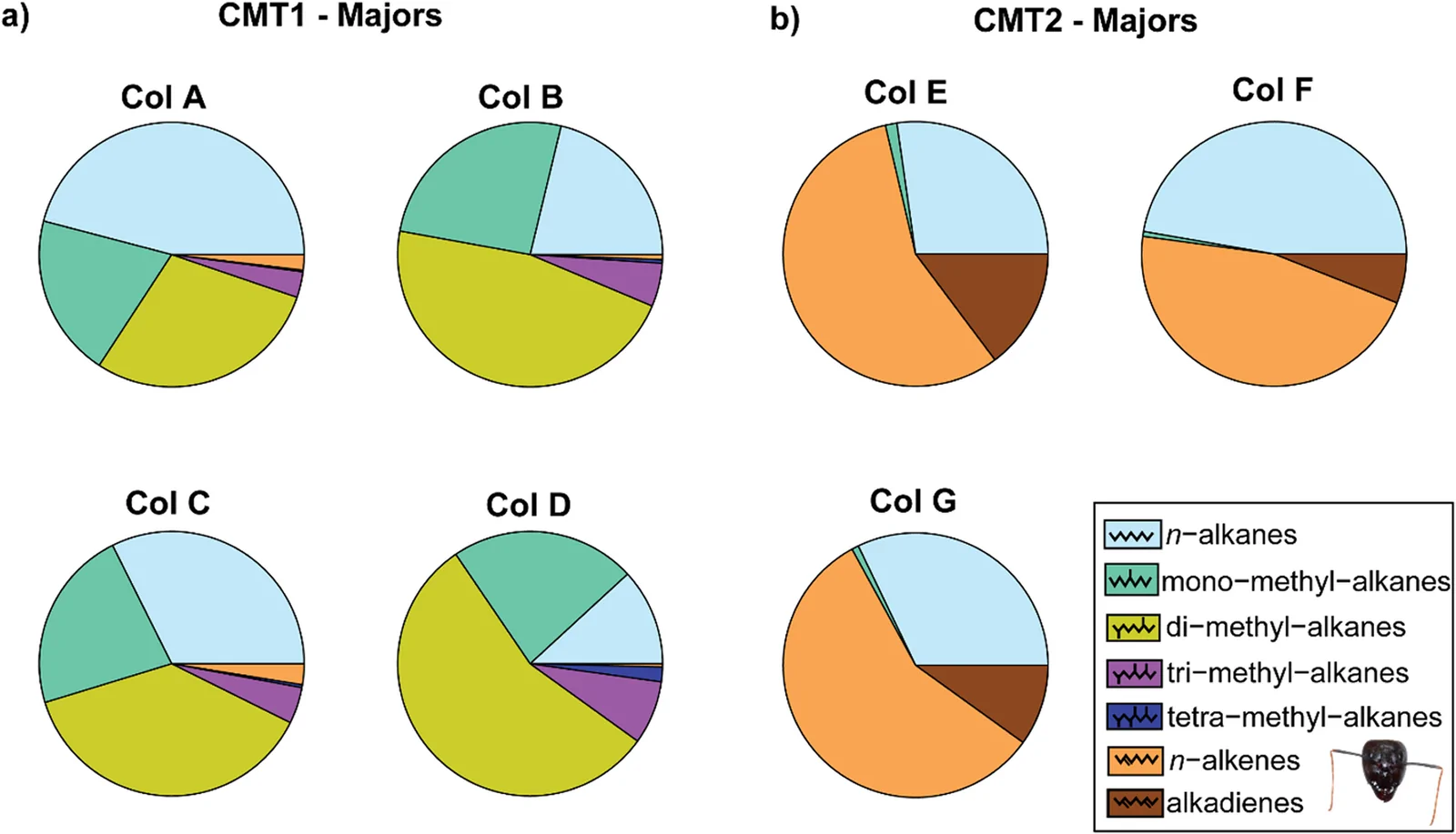
**a** Relative distributions of CHC compound classes detected in major workers from colonies A-D attributed to chemotype (CMT) 1. **b** Relative distributions of CHC compound classes detected in major workers from colonies E-G attributed to CMT2. Relative abundance calculations and assignment of colors for the different CHC compound classes as in Fig. 3

For both minor and major workers of all investigated *C. maculatus* colonies, we observed a distinctive pattern of vastly different CHC compound class distributions clearly distinguishing the two chemotypes (Figs. 3 and 4; Table 2). CMT1 is predominantly characterized by high abundances of mono- and di-methyl-branched alkanes (Figs. 3 and 4a), whereas CMT2 is dominated by unsaturated hydrocarbons, *i.e.*, *n*-alkenes and alkadienes (Figs. 3 and 4b). Methyl-branched alkanes only occur in small trace amounts in CMT2, whereas a reversed pattern is found in CMT1 with almost no unsaturated compounds detectable (Table 2).

**Table 2:** Comparison of relative abundances of CHC compound classes between the investigated ant colonies. Indicated are colony ID, caste affiliation (min: minor, maj: major), chemotype (CMT1 or 2), and mean relative amounts of the detected CHC compound classes including their respective standard deviations per caste and colony

Col ID	caste	CMT	*n*-alkanes	mono-me-alkanes	di-me-alkanes	tri-me-alkanes	tetra-me-alkanes	*n*-alkenes	alkadienes
A	Min	1	42 ± 8.87	22.11 ± 1.46	30.52 ± 6.58	4.48 ± 1.11	0.67 ± 0.27	0.21 ± 0.28	0
A	Maj	1	45.91 ± 1.77	19.88 ± 1.1	28.97 ± 1.38	3.15 ± 0.35	0.21 ± 0.21	1.88 ± 1.15	0
B	Min	1	29.36 ± 8.02	23.59 ± 4.35	37.8 ± 9.26	8.55 ± 7.41	0.49 ± 0.23	0.2 ± 0.14	0
B	Maj	1	21.24 ± 9.09	25.91 ± 1.27	46.49 ± 7.99	5.31 ± 1.36	0.43 ± 0.17	0.62 ± 0.36	0
C	Min	1	25.27 ± 3.89	28.06 ± 2.99	40.78 ± 4.37	5.41 ± 0.79	0.36 ± 0.25	0.12 ± 0.08	0
C	Maj	1	32.36 ± 5.44	22.36 ± 2.88	37.93 ± 3.27	4.45 ± 0.88	0.35 ± 0.24	2.54 ± 1.52	0
D	Min	1	11.91 ± 2.32	23.06 ± 1.25	56.7 ± 0.99	7.78 ± 0.58	0.41 ± 0.03	0.14 ± 0.05	0
D	Maj	1	11.85 ± 2.35	22.64 ± 1.7	55.57 ± 4.31	7.75 ± 1.14	1.8 ± 2.83	0.39 ± 0.49	0
E	Min	2	32.29 ± 7.11	1.88 ± 1.21	0	0	0	52.3 ± 6.38	13.53 ± 3.09
E	Maj	2	27.26 ± 3.75	1.41 ± 0.45	0	0	0	56.56 ± 4.59	14.77 ± 2.26
F	Min	2	40.81 ± 6.6	2.74 ± 0.97	0	0	0	50.69 ± 4.55	5.75 ± 2.01
F	Maj	2	47.27 ± 10.34	0.6 ± 0.21	0	0	0	46.1 ± 8.27	6.03 ± 3.41
G	Min	2	34.32 ± 5.13	1.42 ± 0.37	0	0	0	53.55 ± 5.43	10.72 ± 1.67
G	Maj	2	32.12 ± 7.17	0.85 ± 0.34	0	0	0	57.11 ± 5.7	9.93 ± 2.81

This chemotypic distinction is consistent across all examined colonies and is maintained in both worker castes. While major workers exhibit a significantly higher absolute CHC quantity than minor workers in both chemotypes (Fig. S1), the relative proportions of compound classes remain stable for both worker castes within each chemotype. Notably, *n*-alkanes are the only compound class consistently present in measurable quantities across all colonies, chemotypes, and castes (Fig. S2–S3). A rigorous visual inspection of representative workers from both chemotypes revealed no consistent morphological differences concordant with the chemotypic differentiation.

**Fig. 5:**
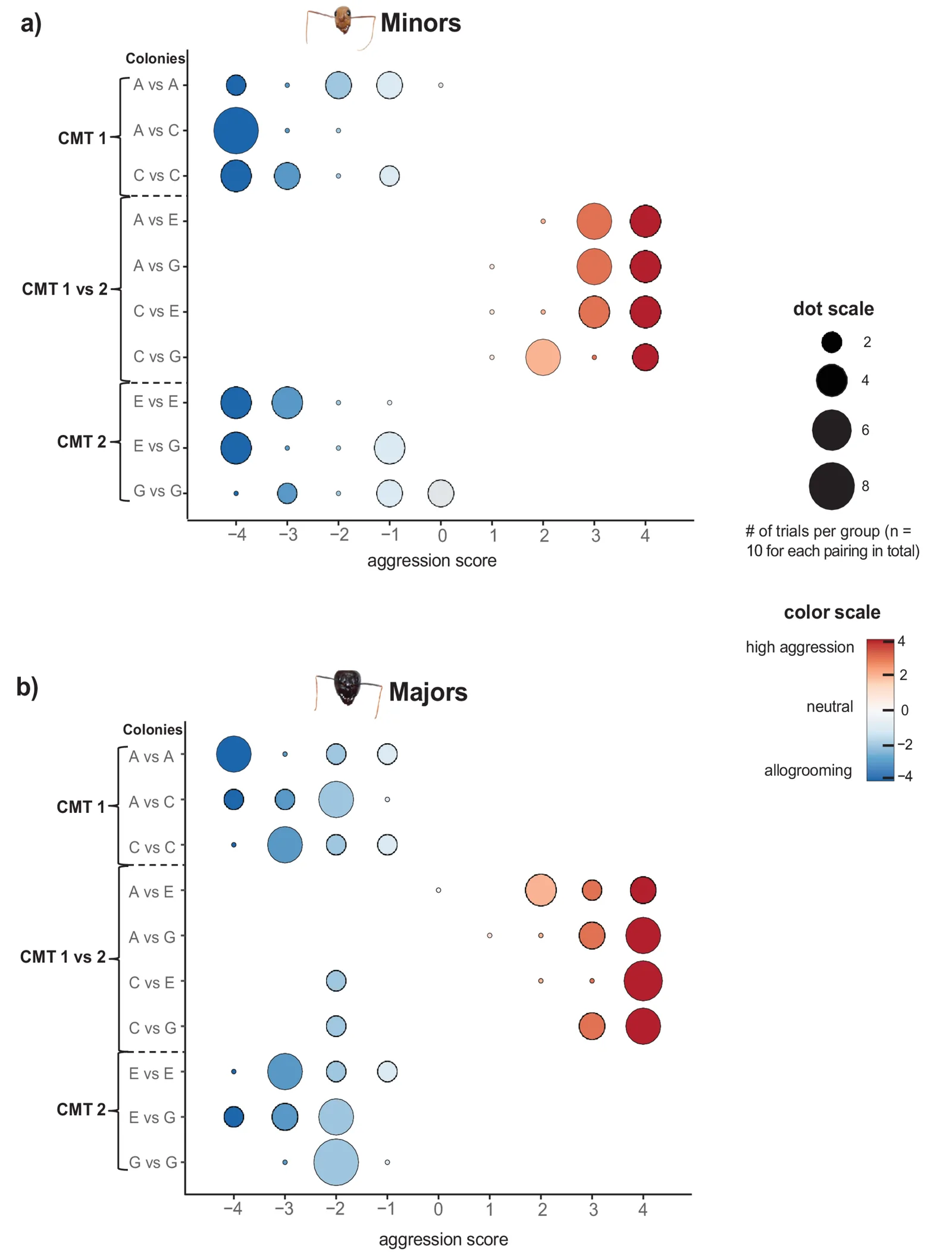
Bubble plots indicating different levels of interaction between minor (a) and major (b) worker pairings from respective colonies representative of both chemotypes (CMT1: Col. A & C, CMT2: Col. E & G). Behavior was scored using a modified version of the scoring table suggested by Krapf et al. 2019. Positive values indicate aggressive behavior and are colored in shades of red, while negative values imply interactions more akin to behavior among nestmates (*e.g*., allogrooming), colored in shades of blue. Dot size corresponds to the number of trials per pairing (n = 10 forg each) that displayed the respective score. The average interaction scores between workers with differing chemotypes were consistently significantly different from the scores between workers of the same chemotype, for both major and minor workers (Benjamini-Hochberg corrected Mann Whitney U tests, p < 0.001)

*Aggression between different Chemotypes.* Aggression assays conducted between individual major and minor workers from colonies representing both chemotypes revealed a clear pattern of chemotype-specific discriminatory behavior (Fig. 5). Workers consistently displayed high levels of aggression when paired with individuals from a different chemotype, indicated by positive values on our interaction scale, for both majors (Avg. 2.7 ± 1.7 SD) and minors (Avg. 3.1 ± 0.9 SD). This is contrasted by consistent levels of affiliative behavior when workers of identical chemotypes were paired, independent of colony affiliation, represented by negative values on the interaction scale in both CMT1 (Avg. -2.6 ± 1.0 SD in majors, -2.9 ± 1.0 SD in minors) and CMT2 (Avg. -2.4 ± 0.7 SD majors, 2.4 ± 1.2 SD in minors). Concordantly, within-chemotype interactions are consistently significantly different from between-chemotype interactions, for both major (M-W, *p* < 0.001) and minor workers (M-W, *p* < 0.001).

Aggressive interactions between chemotypes were frequent and intense in both worker castes, including mandible opening, biting, and prolonged fighting. In contrast, workers of the same chemotype, even from different colonies, not only tolerated each other but frequently also engaged in allogrooming, an affiliative behavior typically restricted to nestmates from the same colony (Esponda and Gordon 2015; Leonhardt et al. 2016; Sprenger and Menzel 2020). This demonstrates strict behavioral discrimination between chemotypes, together with the absence of aggression within chemotypically matching individuals from different colonies.

### Genetic Divergence between Chemotypes

**Fig. 6:**
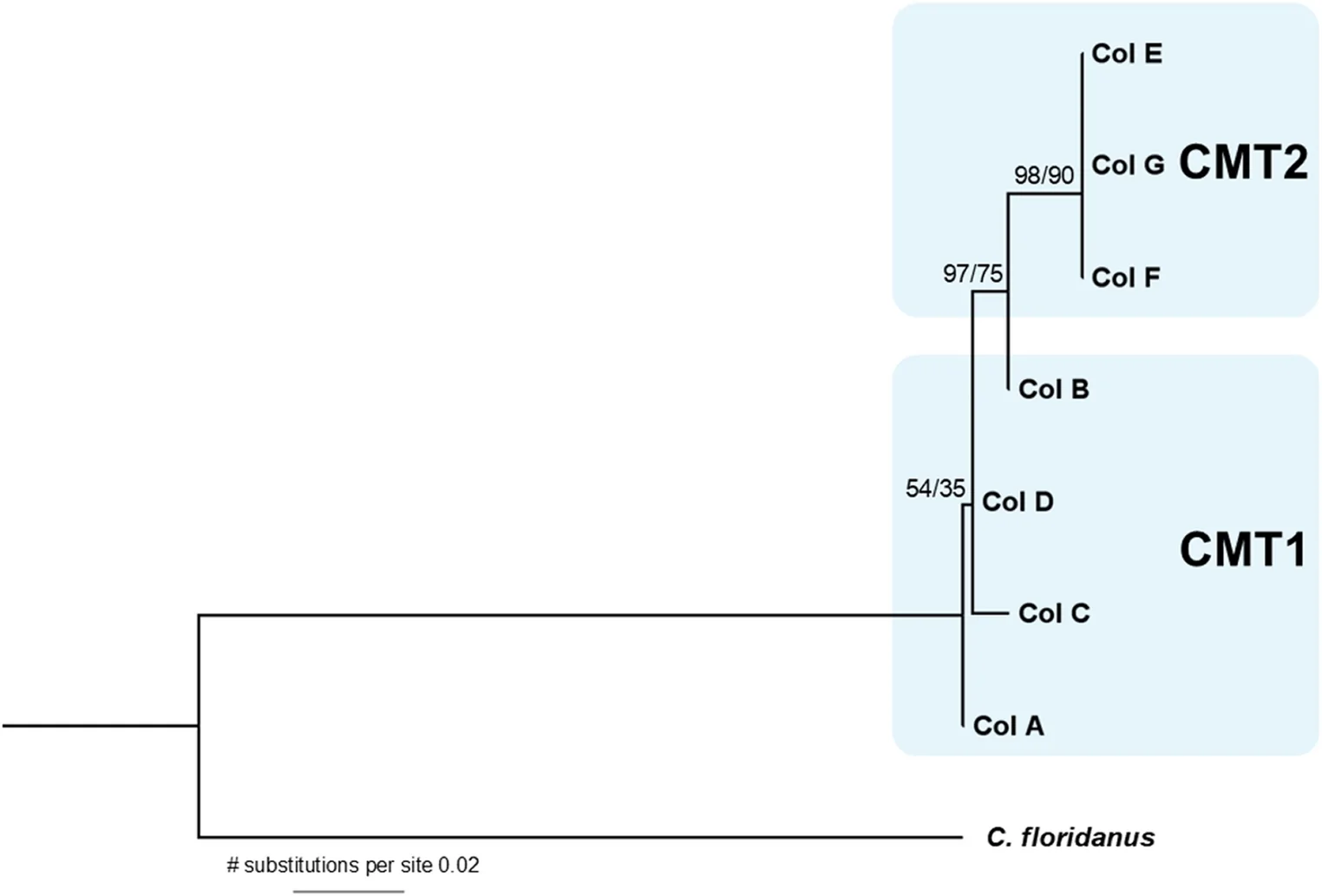
Maximum-likelihood phylogeny of the investigated *Camponotus maculatus* colonies (Col A-F) based on mitochondrial cytochrome c oxidase subunit I (COI) sequences. The tree was constructed in IQ-TREE (v3.1.2) using the TIM2 + F+G4 substitution model (Transition Model 2 with empirical base frequencies and gamma-distributed rate heterogeneity across sites). Node labels indicate SH-like approximate likelihood ratio test replicates/ultrafast bootstrap replicates (SH-aLRT/UFBoot) support values based on 10.000 replicates each. Shaded areas denote the two identified chemotypes (CMT1 and CMT2). *Camponotus floridanus* (AY334397.1) was used as a congeneric outgroup. The scale bar represents substitutions per site

Mitochondrial DNA barcoding based on cytochrome c oxidase subunit I (COI) sequences revealed genetic structuring among the investigated colonies, forming two clades that largely correspond to the previously identified chemotypes (Fig. 6). Maximum-likelihood phylogenetic reconstruction rooted with the congeneric species *Camponotus floridanus* as an outgroup yielded a well-supported singular clade comprising all CMT2 colonies (SH-aLRT/UFBoot = 98/90), while the broader grouping separating the two chemotypes was also strongly supported (97/75). In contrast, genetic relationships within CMT1 colonies were only moderately resolved. Col B diverges the most from the more heterogenous clade associated with CMT1, however, differentiation between chemotypes was still sufficient to recover distinct genetic clusters.

### Caste- and Colony-specific Chemical Signatures

**Fig. 7:**
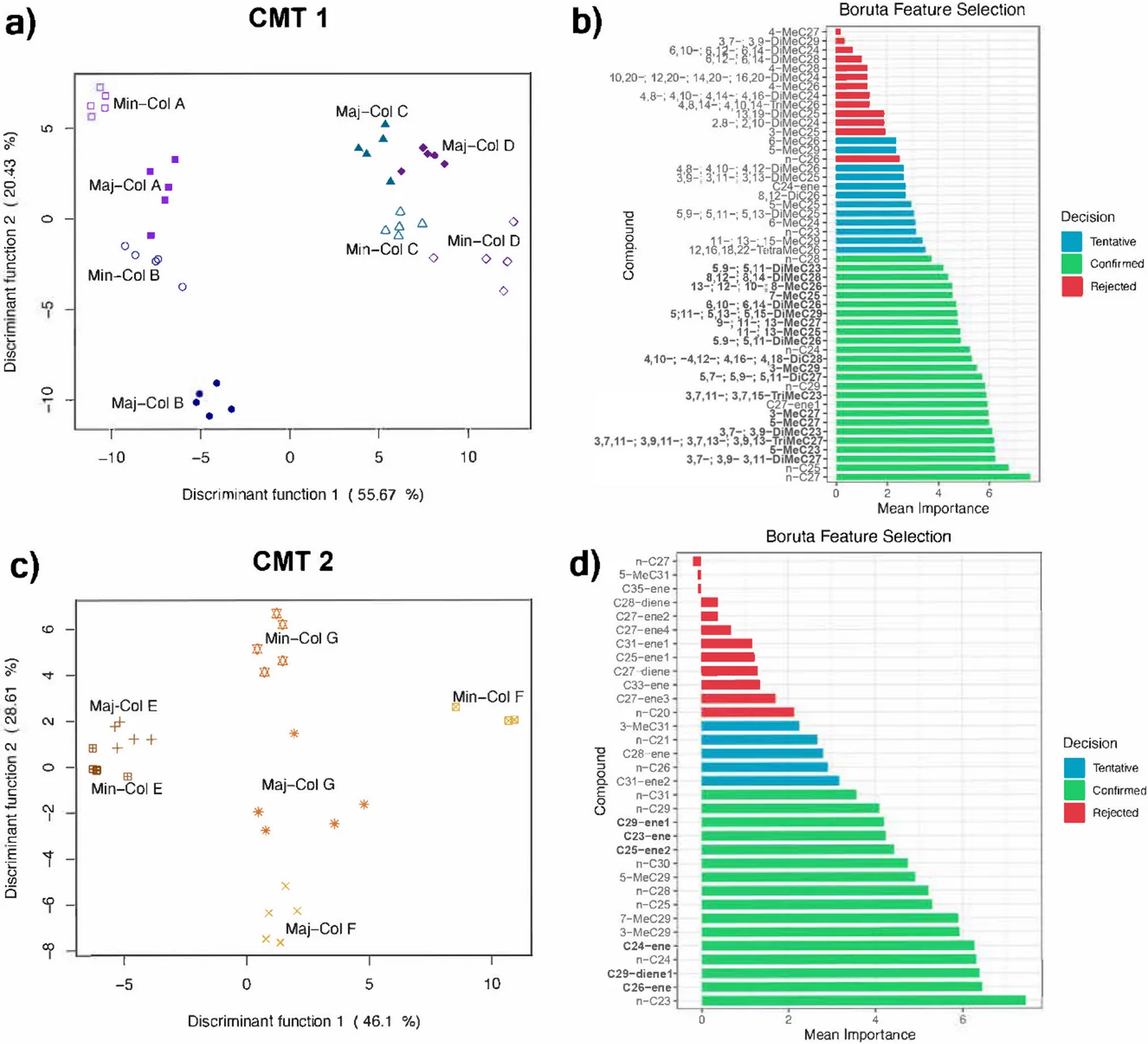
Discriminant analyses (DA) performed separately per chemotype showing the degree of separation between castes and colonies within each chemotype. **a** Chemotype (CMT) 1, consisting of four colonies, shows caste- and colony-specific separation based on CHC profile differences. The two discriminant functions account for 76.1% of the total separation, which significantly separate the investigated groups (Wilk’s λ < 0.0001, *p* < 0.001), colors and symbols as in Fig. 2. **b** Boruta feature selection results showing the individual compounds mostly contributing to the observed separation of groups in the DA (indicated in green for reaching the default mean importance threshold). Mono- and di-methyl-branched alkanes, the dominant compound class for CMT1, are highlighted in bold. **c** CMT2, consisting of three colonies, shows caste- and colony-specific separation based on CHC profile differences. The two discriminant functions account for 74.71% of the total separation, which significantly separate the investigated groups (Wilk’s λ < 0.0001, *p* < 0.001), colors and symbols as in Fig. 2. **d** Boruta feature selection results showing the individual compounds, mostly contributing to the observed separation of groups in the DA. *n*-Alkenes and alkadienes, the dominant compound class for CMT2, are highlighted in bold

Beyond the most obvious separation into the two uncovered chemotypes, we further investigated whether more nuanced chemical differences potentially encode caste- and colony-specific information within each chemotype (Fig. 7). Discriminant analyses conducted separately for both chemotypes based on their internal respective CHC profile divergence revealed clearly separated clusters according to both colony origin and caste identity (Fig. 7a and c).

In CMT1, the first discriminant function which accounted for 55.67% of the total variation primarily reflected colony-level differentiation, with the exception of colonies A and B. Furthermore, the second discriminant function, explaining 20.43% of the variation, effectively separated major and minor worker castes independent of colony affiliation (Fig. 7a). Similarly, for CMT2, colony identity was mainly reflected by discriminant function 1 (46.1% of the total separation), except for major workers of colony G and F. Discriminant function 2, accounting for 28.61% of the total separation, mostly predicts caste-specific separation as in CMT1 (Fig. 7b). This indicates the presence of additional chemical hierarchies beyond chemotype identity.

To identify the specific CHC compounds responsible for this colony- and caste-specific separation, we applied the random forest feature selection model “Boruta”. In CMT1, the separation was primarily driven by the corresponding dominant compound classes,* i.e.*, mono- and di-methyl-branched alkanes, with 19 individual compounds from these classes contributing most strongly to the observed variation (Fig. 7b). In addition, five *n*-alkanes and one *n*-alkene were also identified as minor but significant contributors to caste and colony separation within this chemotype. Conversely, the chemical divergence within CMT2 was driven by a different set of compounds, primarily comprising seven *n*-alkanes, five *n*-alkenes, three mono-methyl-branched alkanes, and one alkadiene (Fig. 7d).

## Discussion

The present study uncovered two sharply contrasting chemotypes separating sympatric carpenter ant populations initially identified as *Camponotus maculatus* into two distinct sub-populations. Through a comprehensive integrative taxonomic approach that combined chemical, behavioral, and genetic analyses, we further solidified the population-specific separation by revealing consistent inter-chemotypic aggression independent of colony affiliation and incipient genetic differentiation. These findings highlight the value of integrating different taxonomic modalities to disentangle morphologically indistinct cryptic lineages.

Ants are among the most ecologically dominant and widespread animal taxa, with over 15,700 described species so far (Bolton 2021; Schultheiss et al. 2022). However, identifying biodiversity in ants is often difficult due to high phenotypic similarity within many taxa, especially when solely relying on morphological characteristics (Seifert 2009; Fournier et al. 2012; Schär et al. 2022). *Camponotus*, in particular, is one of the most species-rich ant genera, with over 1000 estimated species and several hundred subspecies, which are exceptionally difficult to distinguish morphologically (McArthur and Leys 2006; Seifert 2019a). Since morphologically similar species or populations may drastically differ in their genetic (Knaden et al. 2005; Ross and Shoemaker 2005; Schlick-Steiner et al. 2006), as well as chemical signatures (Hartke et al. 2019; Sprenger et al. 2021), these factors should be given more weight in future studies on cryptic differentiation in ants in particular and insects in general.

The pronounced differentiation in cuticular hydrocarbon (CHC) profiles between the two chemotypes is remarkable as it reflects a divergence not only in the relative abundance of individual compounds but also in the predominant hydrocarbon classes (Figs. 3 and 4). Chemotype (CMT) 1 is characterized by a predominance of mono- and di-methyl-branched alkanes, while CMT2, conversely, mostly consists of unsaturated *n*-alkenes and alkadienes. This suggests a shift in biosynthetic pathways and/or selective pressures shaping each chemotype’s chemical signature (Buellesbach et al. 2013; Finck et al. 2016; Sprenger et al. 2021). This is of particular importance given the central role CHCs play in nestmate recognition and colony cohesion in eusocial insects (Sturgis and Gordon 2012; Breed et al. 2015). Furthermore, our aggression assays support the functional relevance of these chemical differences. Workers with the same chemotype, regardless of colony affiliation, never showed any mutual aggression and partially even engaged in affiliative behaviors such as allogrooming and trophallaxis, usually only found between ants of the same colony (Sturgis and Gordon 2012; Esponda and Gordon 2015) These findings suggest that individual workers recognize each another as nestmates based on chemotype-specific cues, underscoring the role of CHCs in mediating social cohesion (Esponda and Gordon 2015; Leonhardt et al. 2016; Sprenger and Menzel 2020). In stark contrast, interactions between workers of opposing chemotypes consistently resulted in high levels of mutual aggression, suggesting the presence of chemically mediated boundaries between the distinct ant sub-populations, independent of colony origin. Interestingly, both major and minor workers exhibited this aggressive behavior, challenging prior assumptions that major workers are the primary agents of inter-colony defense with the most potential for recognition and aggression against non-nestmates (McArthur and Leys 2006; Ferguson et al. 2023). Our results clearly indicate that minor workers also possess the capacity for chemoreceptive discrimination and aggressive behavior when encountering an opposing chemotype (Fig. 5).

Mitochondrial DNA barcoding provides complementary evidence for differentiation among the investigated *C. maculatus* colonies, revealing genetic structuring that was broadly consistent with the observed chemotypic separation (Fig. 6). Intra-chemotype sequence divergence was minimal, while inter-chemotype divergence was statistically robust and sufficient to reflect distinct emerging lineages. This concordance between mitochondrial variation and chemotypic divergence suggests that the pronounced differences in CHC profiles are accompanied by consistent, though relatively low, genetic differentiation.

Given the rapid evolutionary rate of mitochondrial genes, the observed genetic divergence potentially reflects incipient evolutionary separation (Moore 1995; Hebert et al. 2003; DeSalle et al. 2017). This emerging differentiation appears to parallel, and potentially reinforce the uncovered distinct chemical and behavioral separation. This intuitively makes sense, as intricate CHC-based chemical discrimination has long been established as the primary nestmate and species recognition modality in ants that allow colonies, populations and eventually species to maintain their tightly guarded boundaries (Lucas et al. 2005; Meunier et al. 2011; Buellesbach et al. 2018; Van der Meer 2019). Nonetheless, the present phylogeny is based on a single mitochondrial marker gene with a length of approximately 640 base pairs, resulting in a limited number of potentially informative substitution sites. Consequently, the structure of the phylogenetic tree is influenced by only a small number of nucleotide differences. Additional mitochondrial and nuclear gene loci comparisons will be required to further resolve and disentangle the evolutionary relationships among the investigated colonies and assess the extent of genetic divergence associated with the observed chemical and behavioral differentiation (DeSalle et al. 2017).

Furthermore, although less pronounced than between chemotypes, chemical variation within each chemotype indicates additional layers of CHC-based chemical differentiation (Fig. 7). Discriminant analyses revealed that variation within colonies of CMT1 was primarily driven by mono- and di-methyl-branched alkanes, the dominant compound classes defining this chemotype. In contrast, variation within CMT2 was primarily driven by *n*-alkanes, the only compound class occurring ubiquitously in each chemotype, and, to a lesser extent, by the *n*-alkenes that are characteristic of this chemotype. Interestingly, mono-methyl-branched alkanes also contributed to the chemical differentiation within CMT2 despite being present only in small trace amounts. It has been hypothesized that *n*-alkanes have the least potential for encoding chemical information and, conversely, mainly contribute to the waterproofing function of CHC profiles (Gibbs and Rajpurohit 2010; Chung and Carroll 2015). However, several studies have emerged showing that *n*-alkanes can indeed be the main signaling units in different chemical communication systems (Mutis et al. 2009; Lo Pinto et al. 2013; Funaro et al. 2018). Within CMT2, *n*-alkanes might therefore also contribute to the less-pronounced differentiation between worker castes and/or colonies, in addition to the chemotype-exclusive *n*-alkenes. Furthermore, due to the exceptionally high sensitivity of insect olfactory systems, even trace-level compounds such as mono-methyl-branched alkane quantities in CMT2 can potentially play a role in fine-scale chemical differentiation (Sharma et al. 2015; Ghaninia et al. 2017; Fleischer and Krieger 2018). It remains to be seen whether workers also exhibit the capability to differentiate between different castes and colonies within their own chemotype. Addressing this will be pivotal to assess the granularity of CHC-based recognition systems and the extent to which chemical divergence mediates robust caste and colony recognition beyond lineage-level differentiation.

Moreover, it will be intriguing to see in future studies how this profound CHC divergence is governed and maintained genetically. Variations in methyl-branched alkanes have so far been predominantly linked to the activity of fatty acid synthase genes (Chung and Carroll 2015; Sun et al. 2023), whereas the biosynthesis of unsaturated CHCs is largely governed by desaturase genes (Ferveur 2005; Holze et al. 2021). Investigating how these biosynthetic pathways are differentially regulated across chemotypes will be crucial to understand the origin and evolutionary persistence of this remarkable chemical divergence. We hypothesize that the two chemotypes are mainly determined by differences in expression levels of genes from these two biosynthetic families since substantial gene copy number variations between the two emerging sub-populations are rather unlikely given the relatively low genetic differentiation indicated by the mitochondrial barcode marker (DeSalle et al. 2017; Moris et al. 2023). Future studies should also focus on environmental factors shaping these differences, though the initial collection of the investigated ant colonies in close sympatry suggests rather similar ecological conditions. Furthermore, the degree of reproductive isolation between the investigated ant populations remains to be assessed. However, hybridization studies in ants that disperse via mating flights are technically very challenging and have not yet been successfully implemented under controlled conditions (Cordonnier et al. 2019; Seifert 2019b). Nonetheless, exploring whether reproductive barriers exist between chemotypes will be essential to further investigate the evolutionary separation between the indicated emerging sub-populations. Additionally, comparative analyses with other *Camponotus* lineages could provide broader insights and help contextualize the frequency and evolutionary significance of such chemical differentiations.

In conclusion, this study highlights the value of integrative taxonomy in providing complementary insights into diversity and divergence within morphologically conserved lineages. By combining chemical, behavioral, and genetic data, we demonstrate a previously unrecognized divergence within sympatric *C. maculatus* populations, distinguished by their sharply contrasting chemotypes, concordant worker discrimination behavior and associated incipient genetic differentiation. Our findings contribute to the understanding of cryptic lineage diversification and offer valuable insights into the role of chemical communication in potentially driving population differentiation in social insects. As cryptic diversity is increasingly recognized across many taxa, integrative multidimensional approaches such as ours can complement traditional methods by incorporating additional perspectives on taxonomic diversity and the dynamics of ecological and evolutionary divergence.

## Electronic Supplementary Material

Below is the link to the electronic supplementary material.

Table S1: Comparison of absolute quantities of all 73 detected individual CHC compounds averaged per caste and colony. Indicated are CHC compound identifications or possible configurations in case of ambiguities, their mean absolute (ng) amounts as well as their respective absolute standard deviations (sd).

Fig. S1: Average absolute CHC amounts (in ng) from all colonies summarized separately for each worker caste (Maj: major, Min: minor) of chemotype (CMT) 1 and 2. Significant differences were assessed with Benjamini-Hochberg corrected Mann-Whitney U tests and are indicated by different letters.

Fig. S2: Comparison of absolute average CHC amounts (in ng) per compound class in minor workers of each investigated colony. a) n-alkanes, b) mono-me(thyl-branched)-alkanes, c) di-me(thyl-branched)-alkanes, d) tri-me(thyl-branched)-alkanes, e) tetra-me(thyl-branched)- alkanes, f) n-alkenes, g) alkadienes. Significant differences were assessed with sequential Benjamini-Hochberg corrected Mann-Whitney U tests and are indicated by different letters.

Fig. S3: Comparison of absolute average CHC amounts (in ng) per compound class in major workers of each investigated colony. a) n-alkanes, b) mono-me(thyl-branched)-alkanes, c) di-me(thyl-branched)-alkanes, d) tri-me(thyl-branched)-alkanes, e) tetra-me(thyl-branched)- alkanes, f) n-alkenes, g) alkadienes. Significant differences were assessed with sequential Benjamini-Hochberg corrected Mann-Whitney U tests and are indicated by different letters.

## Data Availability

All raw data underlying the manuscript will be made available at the Dryad data repository under [https://doi.org/10.5061/dryad.rbnzs7hq3](https:/doi.org/10.5061/dryad.rbnzs7hq3).
