# Supplementary material for: Sharply Contrasting Chemotypes Coincide with Aggression and Divergence in Cryptic African Carpenter Ant Populations
Source: J Chem Ecol. 2026 Jun 22;52(4):56. doi: 10.1007/s10886-026-01732-4 (PMC13287217; doi:10.1007/s10886-026-01732-4)
Supplement: Supplementary file 2 — Fig. S1: Average absolute CHC amounts (in ng) from all colonies summarized separately for each worker caste (Maj: major, Min: minor) of chemotype (CMT) 1 and 2. Significant differences were assessed with Benjamini-Hochberg corrected Mann-Whitney U tests and are indicated by different letters. [file 10886_2026_1732_MOESM2_ESM.pdf]

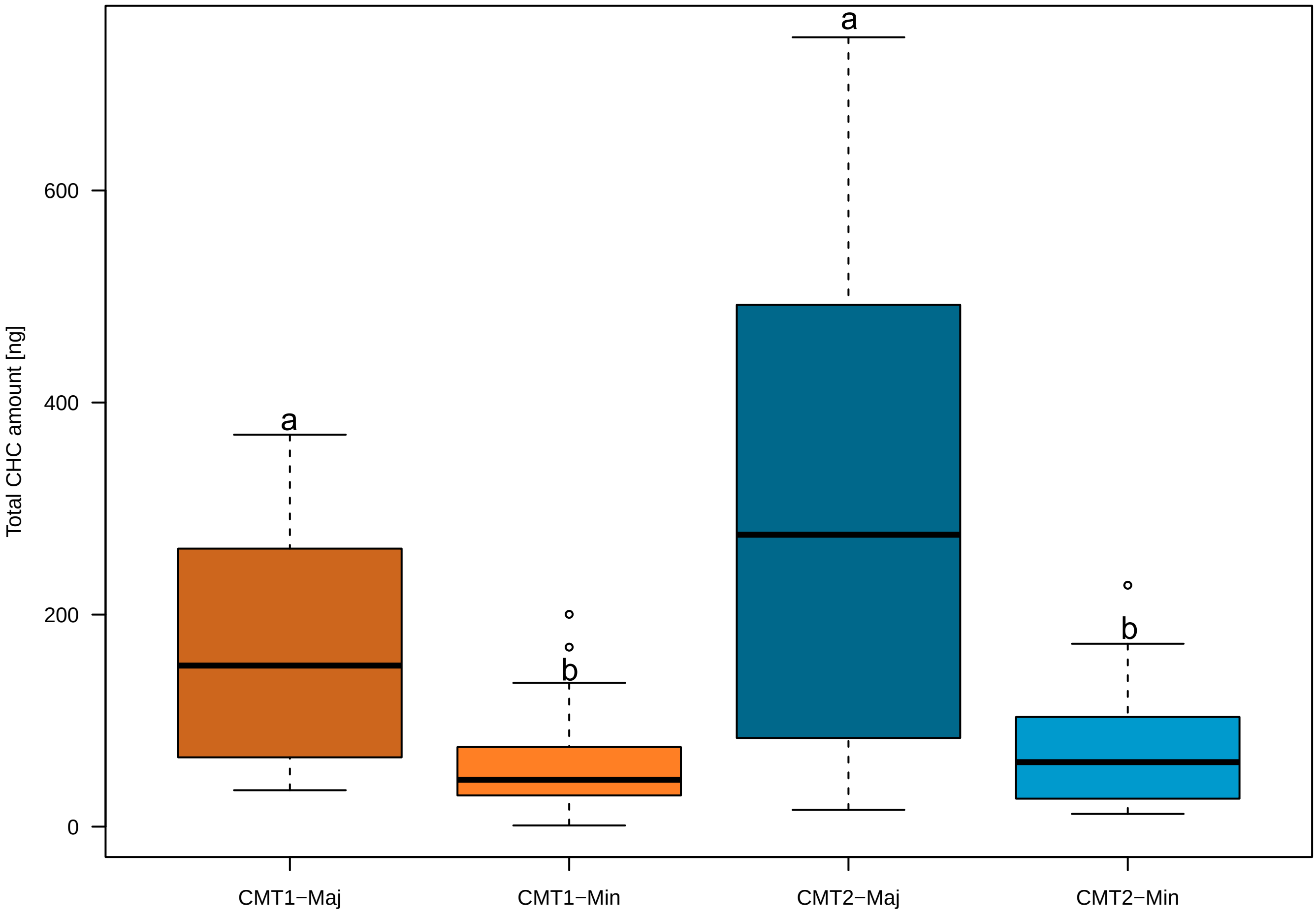


**Fig. S1**: **Average absolute CHC amounts (in ng) from all colonies summarized separately for each worker caste (Maj: major, Min: minor) of chemotype (CMT) 1 and 2**. Significant differences were assessed with Benjamini-Hochberg corrected Mann-Whitney U tests and are indicated by different letters.
