# Supplementary material for: Sharply Contrasting Chemotypes Coincide with Aggression and Divergence in Cryptic African Carpenter Ant Populations
Source: J Chem Ecol. 2026 Jun 22;52(4):56. doi: 10.1007/s10886-026-01732-4 (PMC13287217; doi:10.1007/s10886-026-01732-4)
Supplement: Supplementary file 4 — Fig. S3: Comparison of absolute average CHC amounts (in ng) per compound class in major workers of each investigated colony. a) n-alkanes, b) mono-me(thyl-branched)-alkanes, c) di-me(thyl-branched)-alkanes, d) tri-me(thyl-branched)-alkanes, e) tetra-me(thyl-branched)- alkanes, f) n-alkenes, g) alkadienes. Significant differences were assessed with sequential Benjamini-Hochberg corrected Mann-Whitney U tests and are indicated by different letters. [file 10886_2026_1732_MOESM4_ESM.pdf]

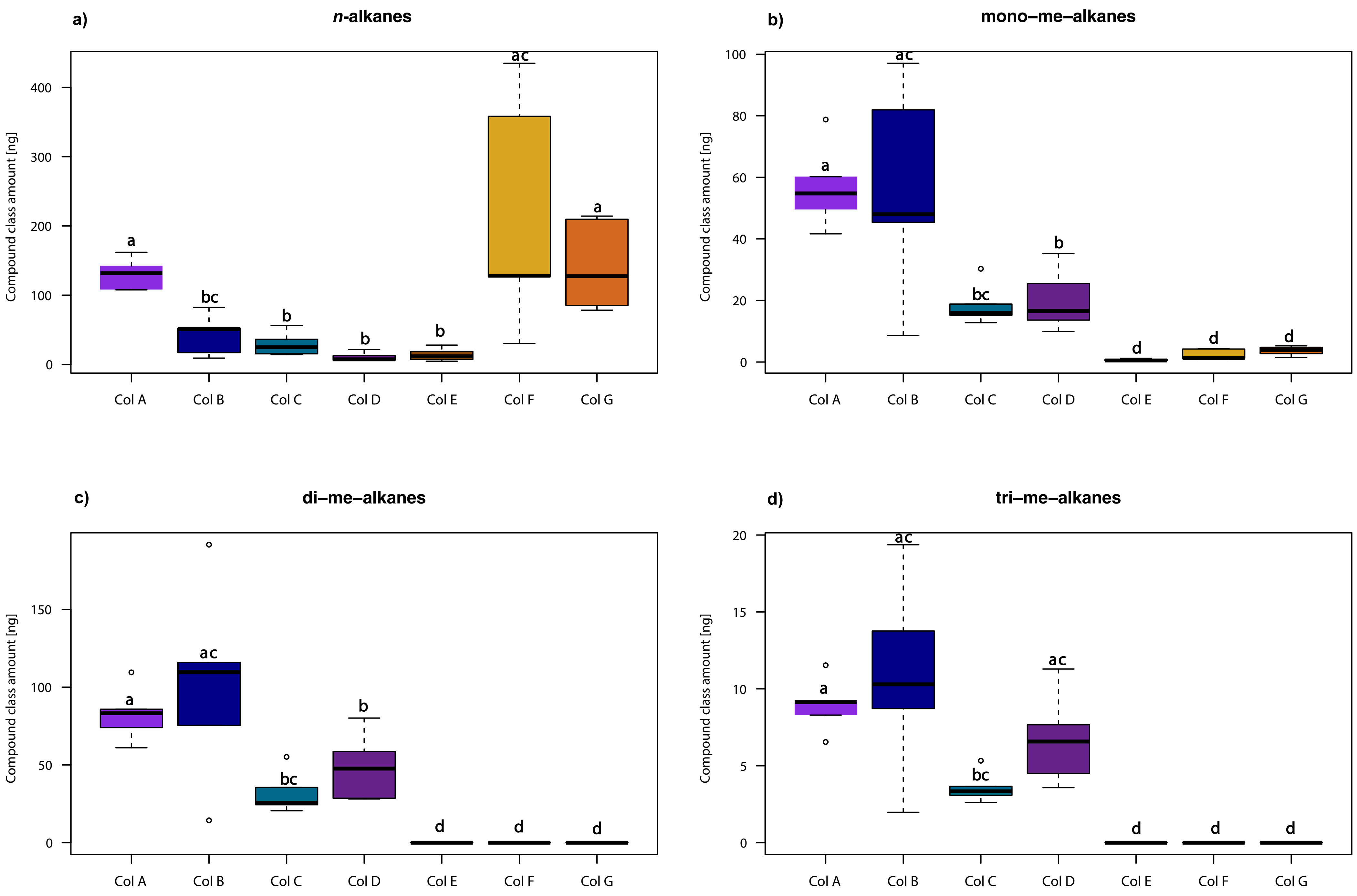
a)
n-alkanes
b)
mono–me–alkanes
c)
di–me–alkanes
d)
tri–me–alkanes
Compound class amount [ng]
Col A
Col B
Col C
Col D
Col E
Col F
Col G
a
bc
b
ac
ac
d
0
100
200
300
400
20
40
60
80
50
150
5
10
15

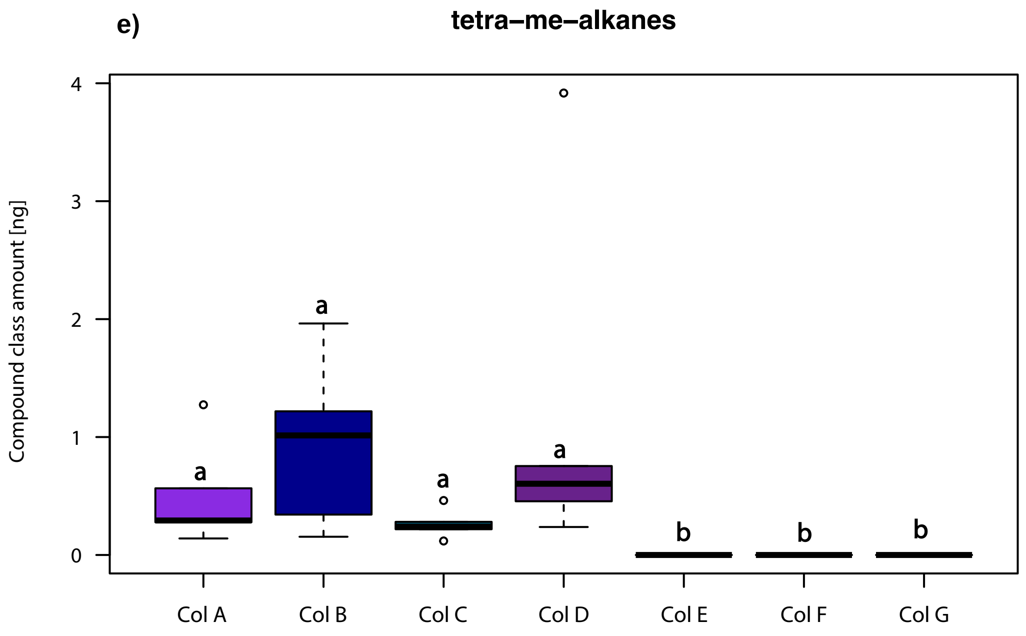

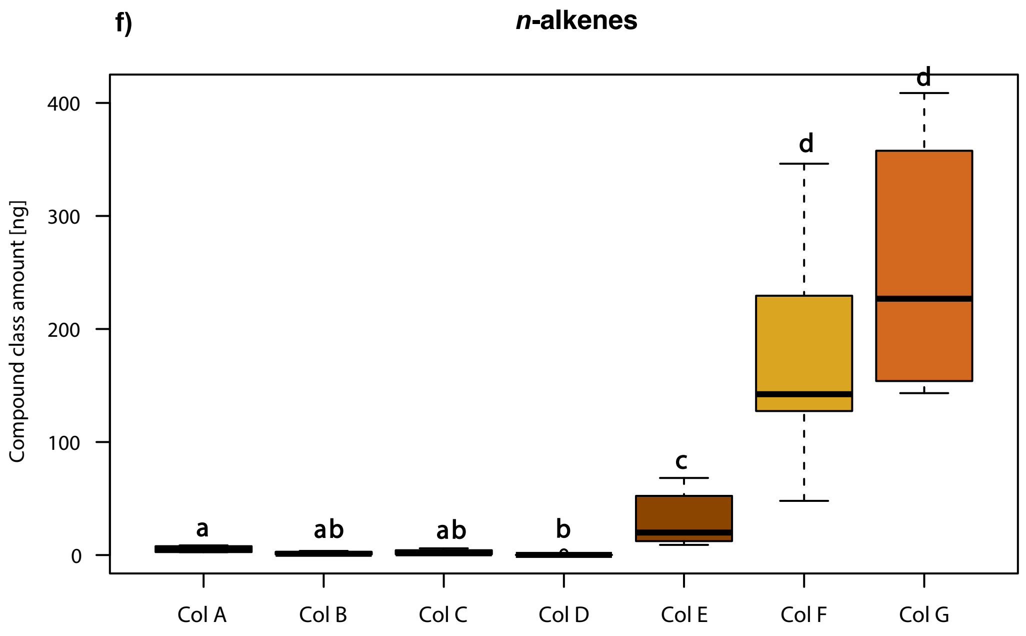

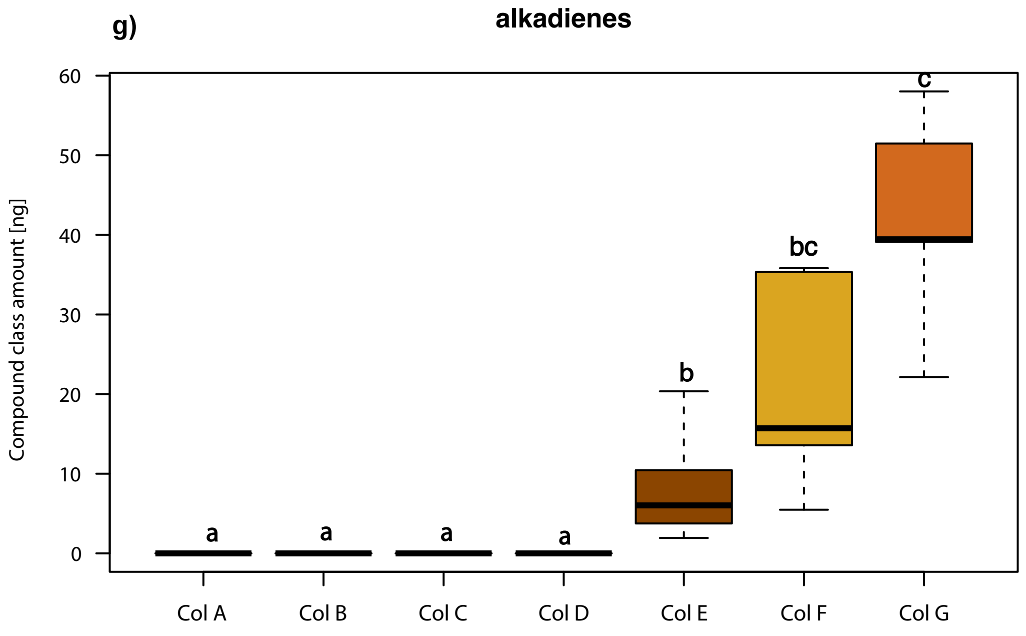

Fig. S3: Comparison of absolute average CHC amounts (in ng) per compound class in major workers of each investigated colony. a) *n*-alkanes, b) mono-me(thyl-branched)-alkanes, c) di-me(thyl-branched)-alkanes, d) tri-me(thyl-branched)-alkanes, e) tetra-me(thyl-branched)-alkanes, f) *n*-alkenes, g) alkadienes. Significant differences were assessed with sequential Benjamini-Hochberg corrected Mann-Whitney U tests and are indicated by different letters.
